# Study of Modified Area of Polymer Samples Exposed to a He Atmospheric Pressure Plasma Jet Using Different Treatment Conditions

**DOI:** 10.3390/polym12051028

**Published:** 2020-05-01

**Authors:** Thalita M. C. Nishime, Robert Wagner, Konstantin G. Kostov

**Affiliations:** 1 Leibniz Institute for Plasma Science and Technology (INP), D-17489 Greifswald (MV), Germany; robert.wagner@inp-greifswald.de; 2 Faculty of Engineering (FEG)—São Paulo State University (UNESP), Guaratinguetá (SP) 12516-410, Brazil; konstantin.kostov@unesp.br

**Keywords:** PET, polymer, plasma jet, tilted application, ROS distribution, UV, VUV

## Abstract

In the last decade atmospheric pressure plasma jets (APPJs) have been routinely employed for surface processing of polymers due to their capability of generating very reactive chemistry at near-ambient temperature conditions. Usually, the plasma jet modification effect spans over a limited area (typically a few cm²), therefore, for industrial applications, where treatment of large and irregular surfaces is needed, jet and/or sample manipulations are required. More specifically, for treating hollow objects, like pipes and containers, the plasma jet must be introduced inside of them. In this case, a normal jet incidence to treated surface is difficult if not impossible to maintain. In this paper, a plasma jet produced at the end of a long flexible plastic tube was used to treat polyethylene terephthalate (PET) samples with different incidence angles and using different process parameters. Decreasing the angle formed between the plasma plume and the substrate leads to increase in the modified area as detected by surface wettability analysis. The same trend was confirmed by the distribution of reactive oxygen species (ROS), expanding on starch-iodine-agar plates, where a greater area was covered when the APPJ was tilted. Additionally, UV-VUV irradiation profiles obtained from the plasma jet spreading on the surface confirms such behavior.

## 1. Introduction

Atmospheric pressure plasma jets (APPJs) have drawn much attention mainly due to their successful application for treatment of materials [1,2,3] as well as biological targets in so-called plasma medicine [4,5]. APPJs are capable of generating chemically rich plasma plumes in open air where ions, electrons, photons, and reactive species are transported to the target allowing the treatment of large and irregular surfaces [6]. They have a great flexibility concerning their construction designs and sizes allowing the generation from micro-scaled jets [7] to plasma plumes up to several centimeters in length [8]. Over the years, many different APPJ configurations have been reported in the literature, such as the ones summarized by Lu et al. [9] where different electrode geometries and excitation sources were highlighted. Depending on the electrode geometry, feeding gas, and power supply, APPJs can be used for a diversity of applications.

Regarding polymer treatment, active species generated in the APPJs are able to interact with only the material’s surface while the bulk is kept unchanged. This characteristic is very important in applications that require biocompatibility and interphase processing, such as painting, dying, and composite manufacturing. During plasma treatment of polymeric surfaces, a series of interactions take place. The first is surface functionalization, where new functional groups are introduced onto the surface due to reactions with reactive species (like oxygen moieties) [10]. The second is surface etching or ablation, in which atoms and molecules of the polymeric chain are abstracted from the surface resulting in the formation of volatile compounds or short polymer chain fragments (low molecular weight oxidized materials) that can be easily washed away [1]. The third is cross-linking at the surface, where two or more parallel polymeric chains are bonded together. This can occur due to either ion bombardment or exposition to UV radiation [1,11]. Thus, after plasma treatment, the surface is modified and can exhibit new properties, such as improved adhesion [12] and wettability [2,13,14,15].

Cold APPJs are commonly applied for the treatment of heat sensitive surfaces, such as thermoplastic polymers [1,16]. Polyethylene terephthalate (PET) is one of the most common thermoplastic polymers used in the food packaging industry [17]. However, it exhibits a chemically inert surface with a low surface energy which leads to poor adhesion properties requiring pre-processing measures. Thus, plasma treatment can help altering the surface characteristics of PET by improving its wettability [12,18,19]. APPJs have been reported as a good alternative surface modification technique where pronounced water contact angle (WCA) reductions of polymers were observed [18,19]. This wettability enhancement was mostly associated with surface oxidation and the formation of polar groups observed by XPS and FTIR analysis [16,18,19]. Onyshchenko and coauthors [19] observed significant changes in oxygen content on a treated PET surface within a circular area with 10 mm radius centered at the plasma plume application point. Vesel and coauthors [18] preformed a detailed study of effective modification zone on PET samples using WCA mappings for different treatment times and distances. They observed that shorter treatment distances allowed the achievement of larger modified areas with lower WCA values while the same surface modifications could be obtained for greater distances, but much longer treatment times. The authors correlated the wettability modifications with the surface functionalization caused by long-living oxygen species.

However, plasma jet treatments usually provide a punctual and localized modification, which is interesting for some applications, but disadvantageous for the treatment of large surfaces and irregular objects. One alternative is the construction of an array or a matrix of plasma jets, where tens of APPJs can be operated in parallel, thus allowing the coverage of larger areas [20,21]. However, it has been reported that the simultaneous operation of many plasma jets can be challenging due to the strong electric repulsion between the neighboring plasma plumes resulting in plasma plume misalignment and, consequently, a non-uniform treatment [21]. Another alternative was reported by Mui et al. [22], where a plasma jet terminating with a horn-shaped nozzle was used for the treatment of PET samples. The 60-mm-diameter conical nozzle allowed a homogeneous treatment over the entire PET sample that lies inside the horn. The uniform modification of PET substrates placed vertically inside the reactor was also demonstrated. Although the obtained WCA reduction extends over a relatively large region, the treatment using such a device relies on the possibility of fitting the object inside the horn nozzle which is not always feasible. For instance, treatment of irregular structures with hollow cavities represents a great challenge. Therefore, a flexible plasma jet that can be used to reach regions of difficult access in the substrate while keeping a small treatment distance would be very important for the modification process. In previous papers we have reported the development and biomedical applications of a bendable plasma jet produced at the end of a flexible plastic tube [23,24,25,26,27]. By any means, the use of flexible devices does not ensure the possibility of keeping the conventional perpendicular arrangement (between the plasma plume and target) as it is commonly used for investigations. Thus, the study of surface modification using different incidence angles of a plasma plume is necessary.

Summarizing the literature data about plasma surface modification of polymers, it can be concluded that the treated area and its radial distribution depend upon many parameters, such as the jet design and power, the distance to the target, the gas flow rate, the treatment time, the plume incidence angle and the target condition (floating or grounded). Most previous works usually investigate the effect of some, but not all, of these parameters together (like plume incidence angle [28,29,30], distance to target [18,19], gas flow rate [31], and treatment time [18,19]) on the target modification process, but very few studies, if none, consider the effect of all parameter. More specifically the effect of target condition being floating or grounded has been frequently overlooked.

In the present work, the modification of PET samples using a malleable plasma jet was carried out. Due to its flexibility, the treatment was performed using different incidence angles between the plasma plume and the PET surface. A similar method to the one used by Vesel and coauthors [18] was adopted here for the WCA measurements. The surface modifications induced by a plasma jet launched parallel to the polymeric surface like in [31] were also investigated. For all studied configurations, the spreading of reactive oxygen species (ROS) on starch-iodine-agar plates and the irradiation profiles of UV and VUV from the plasma plume were correlated with the areas of changed wettability.

## 2. Materials and Methods

### 2.1. Plasma Jet

**Figure 1:**
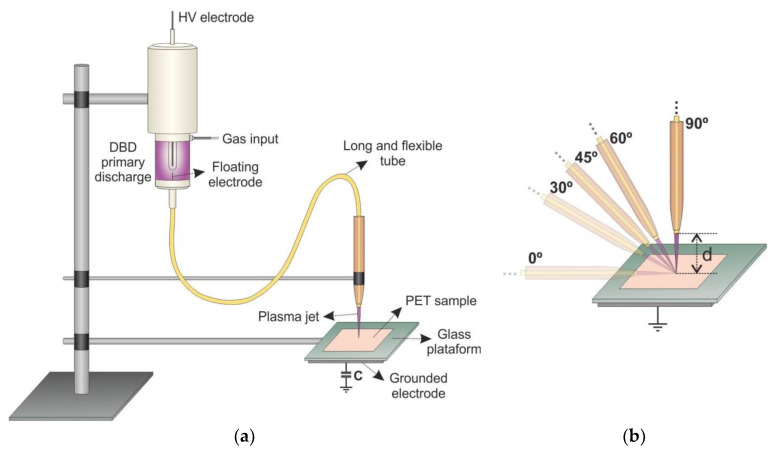
(**a**) Schematic setup of the plasma jet device used in this study and (**b**) arrangement of the plasma plume with different incidence angles. The distance between the tube tip and the substrate is assigned as d.

The plasma source used in this work was already described in previous studies [23,24,25,26,27]. It consists of a dielectric barrier discharge (DBD) reactor connected to a long and flexible plastic tube containing a floating metal electrode inside it. The wire upstream end pops up a few mm into the DBD reactor, while the downstream wire tip terminates a few mm before the plastic tube end. When a noble gas is fed into the primary DBD reactor and high voltage (HV) is applied, plasma is ignited, and the wire tip acquires the plasma potential. The gas is flushed through the polyurethane tube (12 Fr/Ch, Kangaroo^TM^ Nasogastric Feeding Tube, CardinalHealth, OH, USA) and due to the enhanced electric field at the downstream metal wire tip, a secondary (remote) plasma plume can be ejected from the plastic tube end. A schematic layout of the plasma jet device is shown in Figure 1a.

This plasma source was fed with helium with different gas flow rates up to 3.0 slm. The HV electrode was powered by a Minipuls4 AC power supply (GBS Elektronik GmbH, Radeberg, Germany) keeping the same parameters used for the previous biological applications [23,24] (voltage amplitude of 14 kV and frequency of 32 kHz). The voltage signal was amplitude modulated using the burst mode of operation with a duty cycle of 22%, which helps cool down the discharge region, avoiding damage to the plastic tube and treated targets. With a He flow rate of 2.0 slm and the given electrical parameters, the plasma plume has a length of approximately 15 mm. A serial capacitor (1.0 nF) was used for measurement of transferred charge to the grounded substrate.

### 2.2. Sample Preparation and Treatment

Polyethylene terephthalate (PET) samples with a thickness of 0.5 mm obtained from commercial plastic bottles were cut in squares (50 × 50 mm). All samples were cleaned in three steps using an ultrasound cleaner device. First, for 10 min using distilled water and detergent. Second, to remove organic impurities from the surface, they were rinsed with isopropyl alcohol (99.9% purity) for 20 min. Third, the samples were washed for 20 min with only distilled water to remove possible contaminant residues. All samples were dried in a controlled environment at room temperature. After cleaning, the PET samples were glued on glass microscope slides of 1-mm thickness using double-sided tape to ensure a completely flat surface for treatment.

The static PET samples were punctually treated for 60 s of exposure time, with a gas flow rate of 2.0 slm and with different plasma plume incidence angles of 90° (a normal incidence), 45°, and 0°. A schematic drawing of the treatment is presented in Figure 1b. The samples were placed underneath the plasma jet at distances (d) of 5 mm, 10 mm, and 15 mm with different target conditions. In the floating condition, no grounded metal electrode was placed underneath the 5.85-mm-thick glass platform, while for the grounded condition the grounded metal electrode was used. For the sample parallel treatment (0°), the plasma jet was placed 1 mm away from the PET surface and the gas flow rate was varied between 1.0 slm and 3.0 slm.

### 2.3. Water Contact Angle (WCA) Analysis

All treated samples were analyzed by water contact angle (WCA) measurements using the sessile drop method. The WCA measurements were performed in static mode using deionized water with droplets of 0.3 µL. The entire polymeric surface was covered by evenly distributed drops with 3 mm distance within one row and a space of 6 mm in between columns. A similar approach was adopted by Vesel et al. [18]. This method provides a 2D mapping of the treated surface wettability allowing the identification of the actual modification area caused by the plasma jet.

### 2.4. Starch-Iodine-Agar Plates for Reactive Oxygen Species (ROS) Detection

Starch-iodine-agar plates are commonly used as an indicative method for ROS distribution detection from APPJs where the color of reached areas changes to purple. In the presence of water, potassium iodide (KI) reacts with ROS generated in the discharge resulting in formation of iodine (I_2_). This last combines with iodine ions (I^−^) formed from the dissociation of potassium iodide producing triiodide ions (I_3_^−^) [32,33]. Triiodide ions can combine with the starch forming amylose complexes that result in changes of color (purple) enabling the local evaluation of the treated area.

To prepare the plates, 20 g of starch and 25 g of agar-agar were dissolved in 1 L of warm demineralized water. This solution was heated until boiling point and then cooled down to around 80 °C. Only after cooling, 20 g of potassium iodide was added. For experiment execution, Petri dishes were filled with the prepared solution until the starch-iodine-agar layer was around 4 mm thick, which corresponds to approximately 7 mL for 55 mm Petri dishes and around 12 mL for 90 mm ones.

The starch-iodine-agar plates were placed underneath the plasma plume with different distances between the tube tip and the agar surface (1 mm, 2 mm, 3 mm, 5 mm, 10 mm, and 15 mm), for different exposure times and different incidence angles (0°, 30°, 45°, 60°, 90°). In the parallel configuration (0°), the plates were used to evaluate the effect of gas flow variation on the ROS spreading.

After exposition to plasma, pictures of each plate were taken individually under the same illumination, distance, and exposure time conditions. Three different regions could be visualized on the agar surface, however, only the outermost reached zone was considered here. The evaluation was carried out using the free software ImageJ (U. S. National Institutes of Health, Bethesda, MD, USA). All pictures were converted to 32-bit grey scale images and the outermost spreading regions were detected by selecting 94% of the darker pixels within the Petri dish area. Each parameter variation was performed in triplicate. Due to the time delay between plasma treatment and photo shooting, the purple shade of ROS distribution zones cannot be visualized in the pictures presented here.

### 2.5. Ultraviolet (UV) and Vacuum Ultraviolet (VUV) Irradiation Profile Pictures

For a qualitative visualization of UV and VUV effective zones during plasma treatment, phosphor coated plates were used. Based on the fluorescence behavior for specific wavelengths, particular coatings were chosen to detect each kind of radiation. For UV detection a quartz plate with a BaMgAl_10_O_17_:Eu^2+^ coating without binder was employed. The combination of this fluorescent dye and the quartz plate has a spectral sensitivity between 200 nm and 370 nm which allows the irradiation from OH and N_2_ to pass through the coating filter with blue luminescence [34]. The VUV detection device consists of a deposited layer of Zn_2_SiO_4_: Mn also without binder on top of an MgF_2_ optical window (to allow the VUV radiation to be transmitted). Thus, the spectral sensitivity of this device is between 175 nm and 225 nm, which allows the detection of the atomic nitrogen line at 174 nm with green luminescence [35].

For posterior analyses, pictures of the luminescent regions caused by the plasma jet positioning were taken with same illumination, distance, and exposure time conditions. Here, the plasma jet was placed in same positions as for the PET treatment.

## 3. Results and Discussion

### 3.1. Plasma Jet: Electrical Characterization

**Figure 2:**
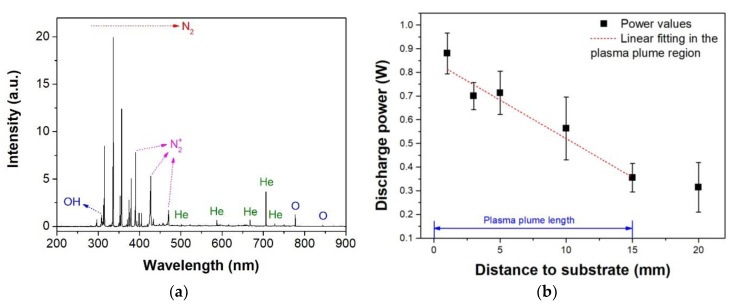
Discharge characterization. (**a**) Emission spectrum of the plasma plume close to the substrate at a distance of 5 mm from the nozzle and (**b**) plasma plume power as a function of the distance between the tube tip and the grounded substrate.

The long tube plasma jet used in this work has already been reported in previous studies [23,24,25,26,27]. In [27], where the same electrical parameters were applied, details of the discharge current and applied voltage waveforms are given as well as specific aspects from the voltage modulation using burst mode with a duty cycle of 22%. Figure 2a presents an optical emission spectrum of the He plasma plume produced at the end of the flexible plastic tube when placed at 5 mm from the substrate with a gas flow rate of 2.0 slm. The spectrum was acquired close to the sample and exhibits a predominance of excited nitrogen emission lines ($N_{2}$- second positive system and $N_{2}^{+}$- first negative system). Ionized nitrogen species are mostly produced by Penning ionization process via He metastables. The presence of excited atomic oxygen and OH confirms the generation of ROS in the plasma plume, which are commonly associated with polymer surface modification [18,19]. Some week emission lines of He can also be observed in Figure 2a.

As already described previously [27], the discharge power from one burst can be obtained from the Q-V Lissajous figure and, for the presented plasma jet, the total power measured corresponds to the power of both discharge regions: primary DBD and secondary plasma plume. For treatments, only the discharge power correspondent to the plasma plume plays a role. In [27], the method used for calculating the discharge power values for each discharge region is explained. Figure 2b presents the plasma plume power for different distances between the tube tip and the PET sample placed on top of a grounded platform. The plasma jet was operated here with a gas flow rate of 2.0 slm and, therefore, had a length of around 15 mm, as indicated in Figure 2b. It can be observed that this plasma jet device works with low power (below 1.0 W) which contributes with avoiding heating damages to the sample. Figure 2b also shows that higher power values were measured for smaller distances. For the region where the plasma plume touches the PET sample (until 15 mm), a linear dependence of the power with distance was obtained, after which no further reduction was detected. This result indicates that the use of a grounded platform influences the plasma jet increasing the power and leading to a more intense discharge.

### 3.2. Normal (90°) Incidence: Distance and Substrate Regime Effect

**Figure 3:**
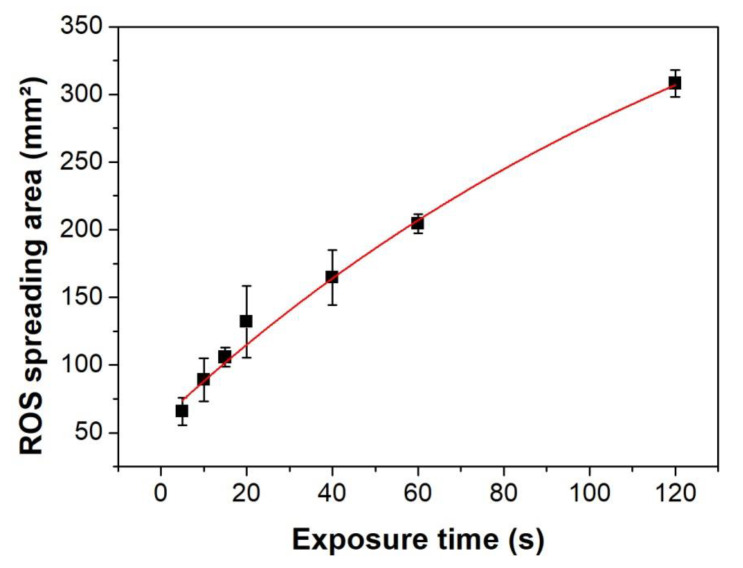
Area covered by ROS on starch-iodine-agar plates for different treatment times and a gas flow rate of 2.0 slm.

Among the processes that promote surface modification in polymers, the attachment of new radicals generated in the discharge also play a role [36]. Such chemical changes and roughness modifications alter the material characteristics and lead to changes in the polymer wettability. Reactive oxygen species (ROS) generated in the plasma plume, e.g., O, O_3_, and OH, are able to diffuse radially where long-lived species, like ozone, can reach longer distances and interact with the polymer surface. Thus, starch-iodine-agar plates were used to identify the maximum spreading area of ROS on the surface due to plasma treatment. Here, the plates were placed at 5 mm from the tube tip (forming 90° with the surface). Figure 3 shows the area variation of spreading ROS when the plasma exposure time is increased (up to 120 s). It can be observed that the affected area on the starch-iodine-agar plates increases with the treatment time tending to achieve saturation. In order to visualize the modified area on PET samples after plasma treatment, an exposure time of only 60 s was chosen. Thus, the treated areas could be easily identified by WCA measurements on 50 × 50 mm polymer samples.

**Figure 4:**
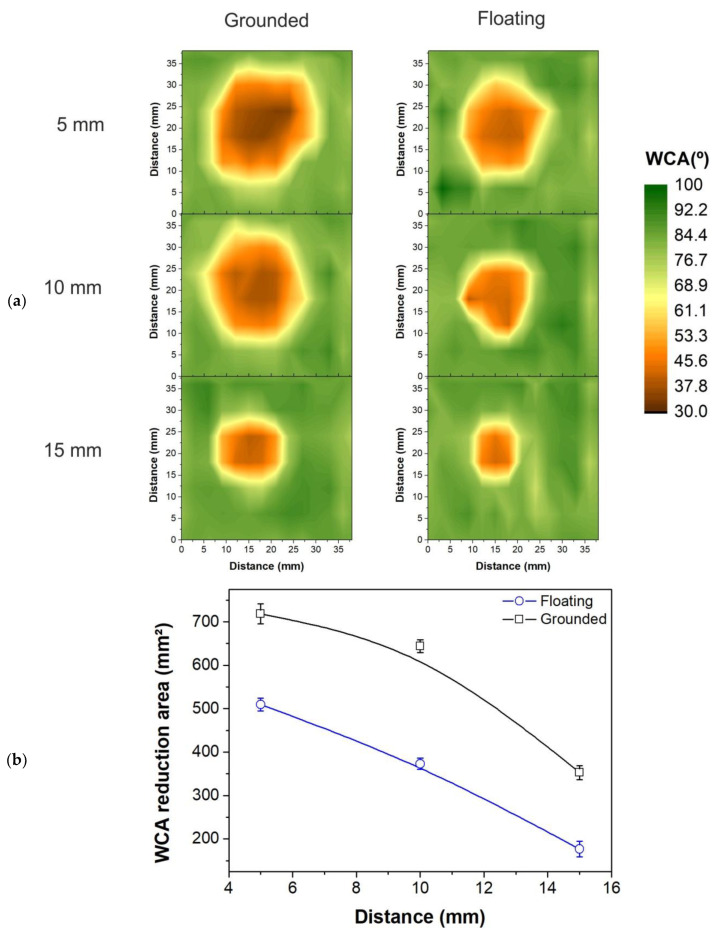
WCA profile of PET samples for a perpendicular (90°) treatment using different distances between the tube tip and the polymer. The treatment was performed under two different substrate conditions: grounded (left column) and floating (right column). The areas of WCA reduction from (**a**) are summarized in (**b**).

In similar conditions, PET samples (50 × 50 mm) were arranged underneath the plasma jet (at the perpendicular position). The polymer was treated for 60 s with a He gas flow rate of 2.0 slm. Figure 4a exhibits the 2D wettability profiles of PET samples measured soon after treatment for different distances: 5 mm, 10 mm, and 15 mm, where the green color corresponds to the untreated regions with WCA of around 90°. In addition, the treatments were performed in two different substrate conditions: floating (right column of Figure 4a) and grounded (left column of Figure 4a). It can be seen that in all cases the modified areas are approximately round, where the major WCA reduction is centered just below the plastic tube exit and from there the WCA values increase radially outwards. Moreover, the treated areas were much larger than the tube diameter (2.5 mm), where a 12-times greater diameter was obtained for the grounded jet at 5 mm (Figure 4a, left column, top map). On the other hand, for the greatest tested distance (15 mm) in Figure 4a for the floating target (right column bottom map), only a six-time greater diameter zone was achieved. The most pronounced WCA drop was observed for the grounded substrate condition, in which case the minimal WCA within the treated area reached about 33° after 60 s of treatment. Besides the lowest contact angle, the grounded jet condition also leads to a more homogeneous WCA distribution.

The area values for all WCA maps from Figure 4a are summarized in Figure 4b. The WCA reduction areas outlines were determined as the regions with around 10% of value reduction (~80°). It is evident in Figure 4b that the use of grounded target condition leads to formation of larger modified areas, where a difference of approximately 30% was obtained when comparing grounded and floating substrate conditions for 5 mm and 10 mm distances. This difference increases to around 50% when comparing both conditions at 15 mm. Therefore, the best results obtained correspond to the grounded target condition for all tested distances. This improvement in achieved area for shorter distances and grounded target are related to the larger treatment dose caused by the increase in power when the discharge is disturbed by the grounded electrode, shown in Figure 2b. Moreover, with shorter distances the plasma species can reach the surface faster and, in case of long-lived species, act for a longer time. The same effect of lesser degree of surface modification induced by the plasma jet at longer distances was observed in a study performed by Vesel and coauthors [18], where PET was treated using seven different distances between 2 mm and 40 mm. Therefore, the data presented in Figure 4 is in good agreement with the WCA behavior observed by these authors.

**Figure 5:**
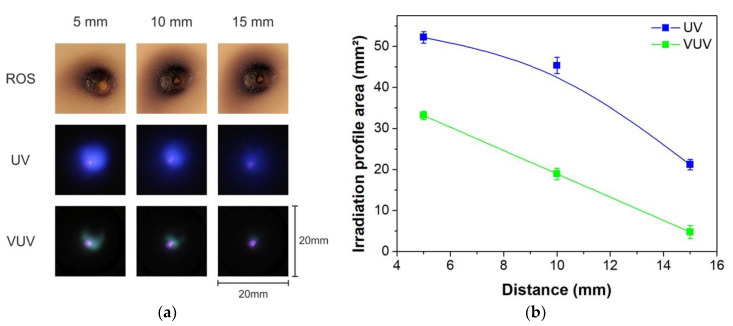
(**a**) Pictures of the affected zones on the starch plates in same scale as UV and VUV irradiation profiles for different distances between the tube tip and the surface and (**b**) calculated areas from the UV and VUV profiles exhibited in (**a**).

ROS are generated in the post-discharge region of APPJs due to air mixing into the plasma plume and they are pointed out as the major agent responsible for PET surface modification [18]. Figure 5a presents pictures of the zones affected by ROS spreading on starch-iodine-agar plates for different distances and grounded platform condition, as performed for the polymer treatment in Figure 4a, left column. In comparison, images of the irradiation profiles of UV (blue) and VUV (green) are also displayed in Figure 5a at the same scale (20 × 20 mm). Differently from the trend observed for the PET treatment, the ROS seem to spread further away from the plasma plume, especially for longer distances. It is important to notice that properties of the two surfaces are very distinct once the starch-iodine-agar plates feature a soft and much more humid surface than the PET polymer, which is solid and dry. Therefore, at shorter distances the He flow deforms significantly the surface creating an indentation (see Figure 5a top line) which, in turn, can perturb the admixture of ambient air into the plume as well as the radial spreading of ROS by the buoyancy effect [37]. In the case of bacteria disinfection on agar plates, Goree and coauthors [38] observed that shorter treatment distances led to a non-homogeneous ring-shaped zone, indicating a non-uniform radial spreading of ROS. Another factor that should be considered for the starch-iodine-agar plates experiments conducted at shorter distances is a discharge intensification with a corresponding plume temperature rise caused by the agar humidity. Furthermore, high temperature and humidity presence are known for hindering the production of ozone, which contributes to the smaller areas observed in the starch-iodine-agar plates test [39]. Thus, due to these important differences between two target surface characteristics, it is expected that the starch-iodine-agar plates experiment cannot be directly compared to the polymer modification at short tested distances once the discharge is intensified and the surface is deformed at this condition. However, for long treatment distances one can expect at least some similarities concerning the size of modified area on both different targets. For instance, for 15 mm treatment, the starch-iodine agar plate and the PET samples exhibit modification areas with similar sizes. Such differences among surfaces is also expected for the treatment of, for example, samples with complex geometries when compared to the flat thin PET substrate, where, for the last, larger modification areas are expected once the plasma species can easily spread on the surface reaching further. Additionally, the treatment of irregular surfaces using a grounded condition might be difficult depending on the sample geometry and its treatment can lead to inhomogeneous modification caused by surface irregularities. However, the use of flat PET samples can be used as a model for surface modification investigations using different processing parameters.

It is well known that UV and VUV radiation generated in plasma jets can cause crosslinking on polymeric surfaces [40]. In He plasma jets, the emissions in UV and VUV come from Penning ionization processes involving admixed air molecules in the plasma plume. As observed in [41] for treatment of PET, the amount of UV radiation produced by a plasma jet is not enough to produce large modifications. However, UV and VUV photons have sufficient energy to break weak bonds at the polymer’s surfaces, thus benefiting oxidation and cross-linking processes [42]. Comparing the sizes of the profiles from Figure 5a, it is clear, that substantial amount of the analyzed plasma agents do not reach as far radial distances as observed for the wettability distribution of treated PET in Figure 4. However, as was already concluded by several authors [18,19,42] the simultaneous action of these active plasma species (even in a small amount) can lead to a strong synergetic effect greatly enhancing the modification potential of the plasma jet. The calculated areas for irradiation profiles of UV and VUV of Figure 5a are presented in Figure 5b. The characteristic size and area of both, the UV and VUV irradiation profiles, tends to diminish when the distance to the plasma plume is increased, as observed for the PET modification in Figure 4b. Additionally, the detected VUV emission for the longest distance of 15 mm is negligible which suggests that, in case of the applied He plasma jet, VUV emission does not play a crucial role.

### 3.3. Incidence Angle Variation: Effective Treatment Area Modification

**Figure 6:**
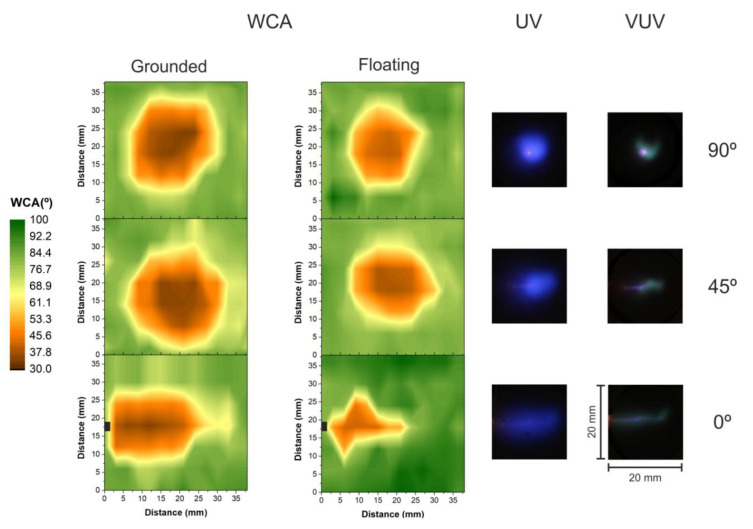
WCA color maps of treated PET for 60 s and three different plasma plume tilting angles, 90°, 45° and 0°, in grounded (left column) and floating (right column) condition. The plasma plume was directed to the right-hand side of the contour maps and the tube tip position is marked in black for the 0° cases. UV (blue) and VUV (green) emission profiles are also displayed for each condition in the same scale as the WCA maps.

In most applications plasma jets are normally kept in a perpendicular position to the target, as for the polymer treatments shown in Figure 4. However, in some cases, like the treatment of irregular surfaces, a manipulation of the plume may be required to reach regions of difficult access. Thus, the remote plasma jet system presented in this study is a good alternative for those cases, allowing an easy handling of the plasma plume enabling the device to be comfortably and safely bent. This manipulation flexibility is also extremely important when the plasma jet is applied in the biomedical field, for instance for treatment inside a patient’s mouth or internal organs. Therefore, the treatment of PET samples and Petri dishes with starch-iodine-agar carried out in this work can be considered as a model study of effective treatment zone variation as a function of the plume incidence angle. To do so, the plasma jet generated at the end of the plastic long tube was directed to the surface using different tilting angles, such as the normal 90° already presented, 45°, and parallel to the surface (0°). Figure 6 shows the 2D WCA mappings for PET samples treated with the plasma jet placed at three different incidence angles for the two different target conditions: grounded platform (WCA left column) and floating condition (WCA right column). The 90° color maps (top line) are the same presented in the Figure 4a for 5 mm distance. For the 45° case, the tube end was positioned 5 mm above the sample center and the plasma plume expands rightward (as demonstrated in the schematic setup of Figure 1b). On the other hand, for parallel plasma plume application (0°) and distances higher than 3 mm, apparently the plasma does not affect the polymeric surface [31]. Thus, in this case, the distance to the PET surface was set to 1 mm, and the tube end was positioned at sample central line close to the sample’s left side as marked in black in the contour maps (in Figure 6 the plasma jet expands rightward). For all three incidence angles, the He flow rate was set to 2.0 slm and the PET samples were exposed to plasma for 60 s.

Comparing the WCA reduction zones for grounding and floating conditions of Figure 6, it is clear that the modified areas for the three different incidence angles treated with grounding condition (WCA left column) were larger than for the floating one (WCA right column). The obtained difference for the normal incidence angle (90°) and 45° was around 30% while, for the parallel treatment (0°), the implementation of a grounded electrode underneath the dielectric platform led to an increase of approximately 60% of the PET modified region. Similarly to the observations in Section 3.2, again the grounding target case led, for all cases of the Figure 6, to the formation of larger and more homogeneous modification regions. For the plasma jet leaning at 45°, a difference in the treated zone shape can be noticed for the two target conditions, where a more elliptically-shaped WCA reduction zone was obtained for the floating platform and a rounder treated region was measured for the grounded case. The shape difference and larger uniform region obtained for the grounding condition were even more pronounced for the parallel (0°) treatment, where a much smaller and completely inhomogeneous modification area was measured for the floating case presented in the right column of WCA in Figure 6.

Tilting the plasma jet for polymer treatment can clearly increase the treated area. The treatments with inclination of 45° (grounded and floating target conditions) presented modification areas around 20% bigger than the ones performed perpendicularly (90°). Another aspect that should be considered is the minimum contact angle value that was achieved. For the grounded condition, the minimum WCA value obtained was around 33°, but no substantial difference in the area of the minimum contact angle was noticeable here between 90° and 45°, while for the floating condition, the treatment led to minimum contact angle value of around 40°, where the 45°-leaning treatment parameter presented a clearly larger zone of the same degree of surface modification. In both cases (grounding and floating) a saturation of the minimum contact angle seems to have been reached. A similar effect was detected by Vesel and coauthors [18] for the increase of treatment time where a wider modification region was obtained with same saturated minimum WCA for longer exposure times. Here, similar effect could be obtained by simply tilting the plasma jet and keeping a short treatment time of 60 s. Comparing the total modified areas on PET samples for the different angles, an increase of around 20% was obtained for the 45°-tilted plasma jet treatments for grounded and floating conditions when compared to the traditional 90° treatment configuration. Damany and coauthors [29] studied the role of the incidence angle of an argon plasma jet for the desorption of organic molecules. The obtained results regarding the affected area are in accordance with the ones presented here. Comparing the tested angles of 90° and 45° to the dielectric surface, the 45° condition was the treatment that led to the wider deposition area of bibenzyl. The authors also observed that tilting the plasma jet and varying the gas flow rate strongly modifies the spatial extension of the developed discharge at the dielectric surface [29].

Further tilting the plasma jet can also be interesting for some applications. Placing a several-cm-long plasma plume parallel to the polymer increases the interaction area [31] and it can help in situations in which the surface needs to be scanned for uniform treatments. For the parallel treatment (0°) presented in Figure 6, the treated area is similar to the one of other two incidence angles (20% smaller than the one obtained for the treatment at 45° but comparable to the 90° case). Additionally, the obtained minimum contact angle is as low as the other tested angles confirming the efficiency of the parallel jet application for surface modification. Differently from the grounded case, the parallel treatment with a floating target condition led to a completely inhomogeneous modification area around 40% smaller than the treatment at 90°. Therefore, the elongated shape of the homogeneous WCA reduction obtained in the grounded condition could be useful for providing a more uniform treatment by one-dimensional scanning of the polymeric surface.

Pictures of irradiation profiles of UV (blue) and VUV (green) are presented next to the WCA color mappings in Figure 6. The scale of all images is the same for better comparison. For the 45° and 0° configurations, the plasma jet tube is leaning to the left-hand side as for the contact angle maps in the left corner in the Figure 6 (also as schematized by Figure 1b). Basically, the UV profiles of Figure 6 present similar shapes to the WCA measured on the treated PET, however they span over smaller areas (the UV distribution being always wider than the VUV one). For the plume incidence angle of 45° the UV distribution exhibits an elliptically-shaped profile that is similar, but with smaller size in comparison to the polymer surface modification. The area of this elliptical profile is around 27% bigger than the profile obtained for the 90° configuration, similar to the increase observed for the WCA measurements. However, the VUV irradiation profiles for 90° and 45° exhibited very peculiar shapes that can be hardly correlated with the WCA patterns. Therefore, the VUV radiation produced by the He plasma jet for distance of 5 mm can be considered as a minor agent in the PET surface modification. However, for the 1 mm distance used for the 0° condition, the VUV emission profile can also be correlated to the modified zone. Thus, the VUV radiation produced by the He plasma jet seems to contribute for PET surface modification only at extremely short treatment distances.

**Figure 7:**
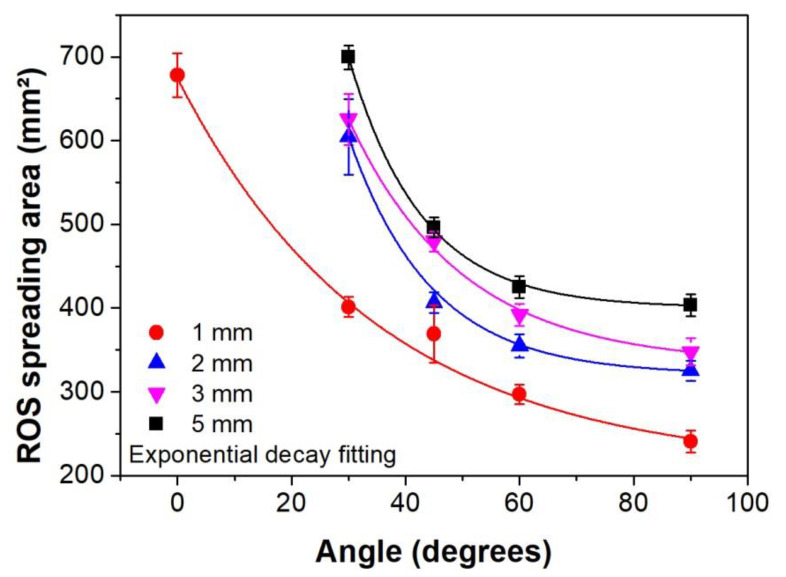
ROS spreading area detected on starch-iodine-agar plates exposed to a plasma jet positioned with different distances (1 mm, 2 mm, 3 mm, and 5 mm) from the agar surface and with different incidence angles (0°, 30°, 45°, 60°, and 90°).

**Figure 8:**
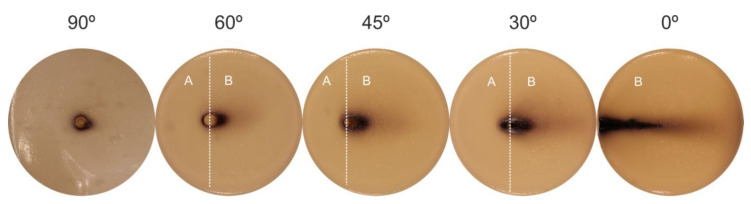
Visualization of the regions on starch-iodine-agar plates (90-mm-diameter) affected by ROS spreading due to plasma jet exposure (60 s) at 1 mm distance with different incidence angles. Here, the plastic tube end is leaning to the left-hand side of the Petri dishes as schematized in Figure 1b. “A” stands for the side of tube inclination and “B” for the plasma plume propagation side. The pictures shown here correspond to the red curve of Figure 7.

Figure 7 exhibits the area on the starch-iodine-agar plates over which the ROS spread for different incidence angles and distances to the surface. Once again, similar behavior like the one in Figure 5 (ROS spreading area increases when the plasma jet is placed further away from the surface) could be observed. From the data presented in Figure 7, it is possible to notice a tendency of rapid area increase when the angle between the plasma jet and the surface is decreased. This trend is in accordance with the area raise obtained for PET treatment and showed by the WCA reduction. The increase in affected area for decreasing incidence angle was observed for all tested distances between 1 mm and 5 mm. For the distances of 2 mm and 3 mm, the affected areas for 0° were very small so that they were not considered. Above 3mm distance, no color modification was observed on the starch-iodine-agar plates for the parallel jet configuration. It is relevant to point out the distinct zone shapes produced on the starch-iodine-agar plates for each jet incidence angle. The pictures of the plates exposed to the parallel plasma jet at 1 mm distance and different incidence angles are displayed in Figure 8 (flexible tube leaning to the left-hand-side). It can be observed that, for the normal jet incidence (90° position), a roughly round figure was obtained. For tilted plume application the WCA patterns gradually evolve into oval-shaped profiles with the rounder part close to the inclination side (side A in Figure 8), that is, close to the lower side of the plastic tube tip. Along the direction of the plasma plume expansion (pointed out as side B in Figure 8), a wider and more diffuse ROS spreading, indicated by the lightest color modification, could be detected. Another interesting observation for the 90° and 60° treatment positions is the formation of a round region (around 4.5-mm-diameter) just where the plasma plume touches the agar, in which no color change was detected. The formation of these ROS-depleted regions at short distance and close to normal incidence can be explained by a strong backward He flow produced by gas reflection from the surface, which causes a blocking effect to the surrounding air molecules. They are hindered from diffusing into the effluent, leading to a drastic reduction of ROS formation. Starting from 45° jet inclination this effect is strongly reduced and probably an efficient gas mixing is obtained in the plasma effluent. The shape of ROS affected area evolves from an oval to elliptical pattern for the 30° treatment. In this case a darker spreading zone, when compared to the larger angles, suggests that more ROS are produced and they are able to propagate further. Finally, the parallel position of the plasma jet (0°) led to formation of a very elongated ROS pattern in a shape similar to the plasma plume. The shapes of spreading reactive oxygen species can be correlated to the ones obtained in the WCA analysis and to the UV irradiation profiles for 0°, 45°, and 90°.

### 3.4. Parallel Treatment (0°): Gas Flow Variation

The easy handling characteristic of this plasma jet device can be especially interesting when it comes to surface modification and deposition once these applications require short distances in order to obtain good results. A good example of treatment of irregular objects is the modification of the inner surface of plastic tubes for surface activation studied by Prysiazhnyi et al. [3]. However, as mentioned above, for surface scanning purposes, applying the plasma plume in parallel can be advantageous and then, in this case, adjustment of the applied parameters is necessary. In this configuration (0° between the plume and the surface), regulating the gas flow rate can lead to a more homogeneous modification and also to larger treatment areas. In [31] the authors investigated the surface modification of polystyrene (PS) by side-on plasma jet under the floating target condition and found that the gas flow rate was an important parameter. The data in Figure 4 and Figure 6 show that the grounded target condition always led to better surface modification (wider treated area and lower WCA). Moreover, the comparison between floating and grounded conditions for the parallel treatment (0°) showed a considerable difference of 60% larger and a more homogeneous surface modification area for the grounded case (Figure 6). Thus, all further treatments using parallel plume application were made in this condition (grounded).

**Figure 9:**
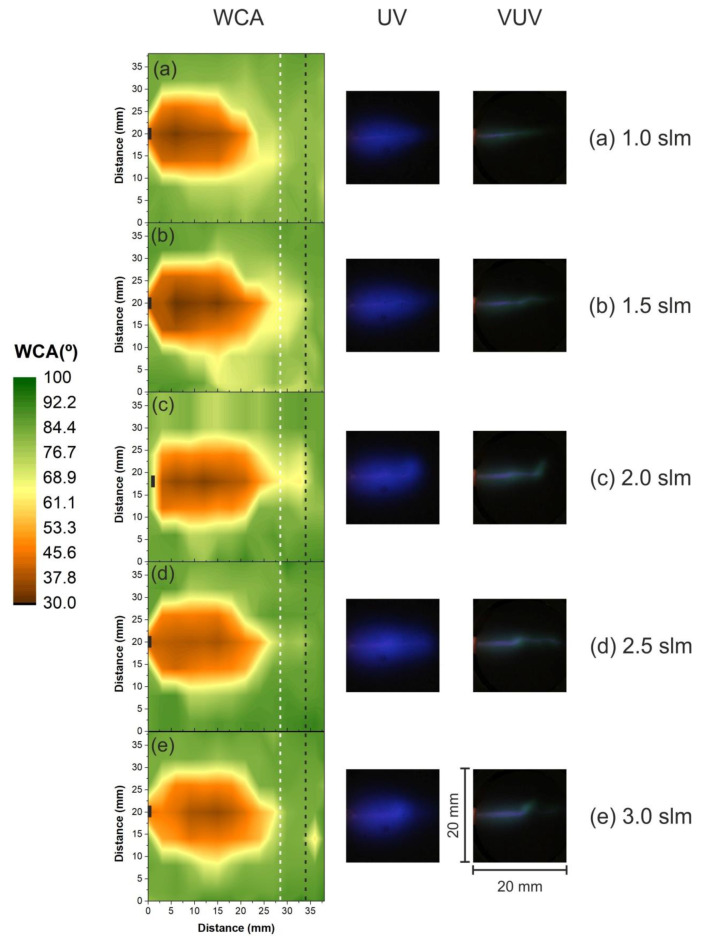
WCA reduction color map of PET samples treated by a He plasma jet placed parallel to the polymer surface for different applied gas flow rates: (**a**) 1.0 slm, (**b**) 1.5 slm, (**c**) 2.0 slm, (**d**) 2.5 slm and (**e**) 3.0 slm. The black rectangle identifies the position of the tube tip. UV (blue) and VUV (green) emission profiles are also displayed for each condition in scale.

The 2D WCA mappings for the parallel (0°) treatment of PET samples are presented in Figure 9. Each letter in this figure corresponds to a different He gas flow rate from 1.0 slm (a) to 3.0 slm (e) in ascending order. To compare with the contour maps, pictures of the UV and VUV irradiation profiles are shown at the same scale. In all pictures exhibited in Figure 9, the parallel plasma plume is directed to the right, where the position of the plastic tube end is marked by a black rectangle in the WCA maps. Olabanji and Bradley [31] reported the presence of untreated zones close to the plasma jet nozzle for high gas flow rates when treating PS in the floating condition. Differently from what they observed, in all cases the area of the PET samples modified by the plasma plume and grounded condition has an elongated (roughly elliptical) shape that starts at the plastic tube end and extends rightward. This difference might lie on the different substrate regimes studied in each case (floating target in [31] and grounded substrate here). Observing Figure 9 it is evident that the longitudinal extension of the surface modification varies with the gas flow rate, while its transversal size stays more or less unchanged. The white dotted line on top of the WCA contour maps shows the limit of the treated length for the 1.0 slm condition. It is clear that the length of the modified area increases until the gas flow rate of 2.0 slm (indicated by the dashed black line) after which it starts decreasing. Such a decrease in the modification length of the polymer surfaces for the increased gas flow rate was also observed for Olabanji and Bradley [31]. A similar trend is observed for the treated areas whose values increase approximately 15% when raising the gas flow rate from 1.0 to 1.5 slm or 2.0 slm. For pure helium flow, in such conditions, the obtained Reynolds number is quite low: Rn between 73 and 217 for 1.0 slm and 3.0 slm, respectively. The obtained values are also much lower than the critical Reynolds number of Rn = 2000 that indicates the laminar-turbulent transition [43]. Thus, the He flow is expected to be laminar for all chosen conditions. However, the target presence in the close vicinity to the He gas flow may affect it. Additionally, with plasma on, the fluid dynamics change completely, and a turbulent condition can be much more easily achieved. It was observed by Winter and coauthors [44] with shadowgraph images that a laminar He flow from a plasma jet device can become turbulent when the plasma is ignited. Thus, the shortening of the plasma plume length above 2.0 slm can be explained as the gas flow transition from laminar to turbulent regimes. Indeed, the transition seems to occur at 2.0 slm, where even though the modification length and area are as large as for 1.5 slm, an extra region of low WCAs can be observed further away along the jet axis. This pattern can also be observed in Figure 9c–e (2.0 slm, 2.5 slm, and 3.0 slm) and it arises from the unstable plasma plume typical for turbulent flows. Thus, the largest areas of lower contact angle values correspond to the laminar conditions (1.0–1.5 slm) and to the transition from laminar to turbulent flow (2.0 slm), where a reduction of WCA to almost 30° was obtained. The five PET modification areas presented in Figure 9 exhibited a similar plume-like longitudinal shape. This pattern can also be observed for the UV and VUV radiation profiles, where the transition to turbulent mode for 2.0 slm is defined by the diffuse plasma jet tip observed in both cases. Differently from the previous experiments (Section 3.2 and Section 3.3). Here the VUV profile matches the observed modification on the PET samples. Therefore, it is expected that the VUV radiation generated at the plasma plume plays a role only for very short distances (as in the case of side-on plasma jet application). For larger jet-to-target separations, as in the previous sections, the generated VUV is efficiently absorbed by the ambient air [34,41].

**Figure 10:**
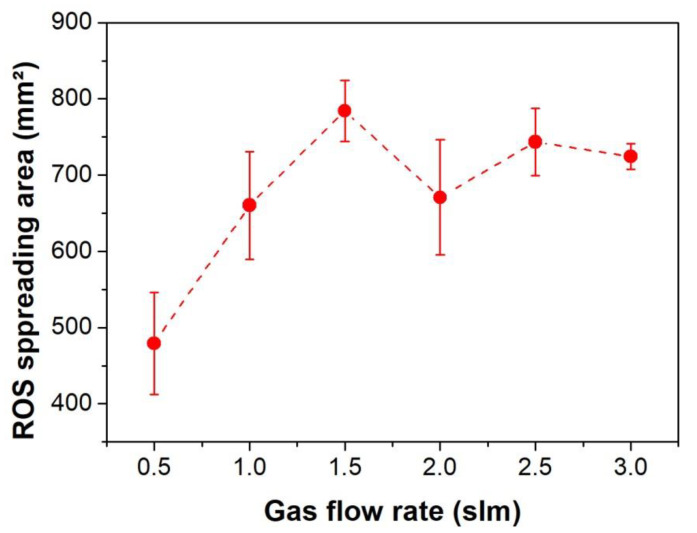
Effective spreading of ROS on starch-iodine-agar plates when exposed to plasma at 1 mm distance in parallel (0°) configuration for 60 s.

The same investigation as in Figure 8 was done for detecting the ROS spreading on starch-iodine-agar plates with the plasma jet in parallel with the agar surface at 1 mm distance and varying the He flow rate. The results are presented in the Figure 10 for different gas flow rates between 0.5 slm and 3.0 slm. The area of spreading ROS tends to steadily increase up to 1.5 slm. From 2.0 slm the ROS covered area is slightly reduced due to the gas flow transition from laminar to turbulent mode in accordance to the observations of the WCA measurements and UV/VUV profiles. Thus, for achieving the largest treated area with more homogeneous modification, the operation of the plasma jet at 0° should be within the laminar flow regime (up to 1.5 slm in this case) and operating in the grounded condition. Since the highest concentration of reactive species is close to the effluent region, the plasma jet operation in laminar mode, at 0° and 1 mm distance, appears to be the most attractive configuration for PET treatment in which most of the produced plasma species generated along the entire plasma plume extension can act on the surface leading to a more efficient WCA reduction within a short treatment time.

### 3.5. Combination of Different Processing Parameters: Treatment Optimization

Variation and combination of all the processing parameters presented previously make the surface modification by plasma a complex process. It is difficult to find a specific set of parameters that would lead to the best surface modification results once some of these parameters are not independent. For instance, increasing gas flow rate (within the limit of laminar flow) makes the plasma jet length increase, in this way, also affecting the jet-target distance. On the other side, the target presence can also affect the transition from laminar to turbulent flow. Moreover, in most cases the surface modification effect does not scale linearly with any of these parameters. For instance, the active species produced in the plasma plume have limited lifetimes and the increase of treatment time, especially at larger distances, does not result in a lower WCA. Additionally, in general, the treated area increases with decreasing the distance. However, at very short distances, gas turbulence and buoyancy effects affect the ROS distribution and hinders any further increase of the modification area. Therefore, choosing a very short distance and a high flow rate does not necessarily mean the best treatment condition (i.e., the lowest WCA for the shortest treatment time). Thus, to achieve the best surface modification results (including a large and more uniform modified area) the selection of an appropriate set of gas flow rate and jet-to-target distance are important. This depends upon some other experimental parameter like the kind of gas used (He, Ar, or some other), the jet exit dimension, the target surface condition (soft or solid), the ambient humidity and, of course, the particular treatment goal (a punctual or large surface area modification). These will establish the primary set of parameters for an optimal treatment. In this study, we focus on two additional and sometimes overlooked parameters—the jet incidence angle and the target grounding—which can also affect the surface modification effect, significantly increasing (in some cases by about 20%) the size of modification area. Their variation can establish a second order of process optimization. According to the presented study, in order to obtain a more efficient surface modification with a shorter treatment time, the use of incidence angles smaller than 90° combined with a grounded target condition are important. Finally, the jet incidence parallel to the target is an interesting case that needs further investigation because it seems to be very much affected by the target grounding condition.

## 4. Conclusions

The wettability of PET samples after plasma jet treatment, conducted under different conditions was investigated using the WCA mapping method, which allowed the detection of the entire modification zone. This study allowed identifying two important alterations in the experimental setup configuration that led to a larger treated area and enhanced treatment homogeneity: the use of a grounded substrate holder and the effect of different jet incidence angles. The latter was shown to promote a better ROS spreading on the surface, which contributes to increasing the modified area on PET samples and leads to the formation of larger zones of more efficient treatment. This feature can be especially interesting for applications like the treatment of polymeric packaging, thin films deposition, and biomedical applications where the treatment of regions with difficult access may be required.

For all studied cases, the evaluations of ROS and UV spreading were in reasonable agreement with the WCA measurements. Thus, the effective area of modification can be correlated to the activity of different plasma components that work synergistically. For the shortest tested distance of 1 mm, in the parallel jet arrangement, the VUV irradiation produced by the plasma jet also seems to play a role.

The spreading area of ROS over the starch-iodine-agar plate changes drastically when the angle formed between the plasma jet and the treated surface is diminished. Its shape varies from round (at 90°) to elliptical (at 30°), and finally to a more diffuse and elongated plume-like shape (0°). Thus, according to the desired application, the choice of a particular incidence angle can provide better results, being able to adjust the treatment from more punctual to rather diffuse.

Applying the plasma jet parallel to the surface has shown to be the most attractive condition because a relatively large and homogeneous modification zone can be obtained in the longitudinal direction, which means that a large sample can possibly be scanned only in one direction (transversal to the plasma plume). The variation of gas flow rate can be used to adjust the size of the modified area where the transition from laminar to turbulent plasma plume can be easily controlled. Additionally, simply adjusting the incidence angle of the plasma jet can efficiently vary the resultant modification areas allowing an advantageous control over the treatment for different desired applications.

In conclusion, surface modification of materials by plasma is a complex process where several processing parameters, such as, jet-to-target distance, gas flow rate, treatment time, target condition, and jet incidence angle, should be considered in order to control the resultant modification area and efficiency.
